# Hospital and Institutional News

**Published:** 1921-03-05

**Authors:** 


### HOSPITAL AND INSTITUTIONAL NEWS.

### THE HOSPITALS INQUIRY PROGRESS.

Lord Cave's Committee is continuing to hear evidence at its Wednesday meetings from representatives of London hospitals and medical organisations. In addition to those mentioned in our last note, Mr. Thomas Hayes, clerk to St. Bartholomew's Hospital, has been called, and Mr. Gilbert Panter, secretary of the Great Northern Central Hospital. On Wednesday, February 23, representatives of the British Medical Association were heard, and representatives of the Royal College of Physicians, King's College Hospital, the London children's hospitals, and the London women's hospitals were invited to attend last Wednesday. We are glad to notice that the provincial hospitals are bestirring themselves through the Regional Committees of the British Hospitals Association so as to be prepared with representatives to attend this Committee when the question of provincial hospitals is before it.

### THE HEALTH MINISTRY AND PAYING PATIENTS.

The Ministry of Health wants to know more about schemes for taking paying patients proposed to be instituted by Poor-law guardians at Manchester and elsewhere. Such schemes, the Ministry considers, raise very important principles, medical and other, and a fuller statement of the nature of the proposals at Manchester is asked for stating what, if any, steps have been taken towards bringing the scheme into operation. However, it is evident that at Manchester the Board of Guardians acts first and asks permission afterwards, for the scheme came into operation on February 1. It is not stated what work has been done yet, whether progress in the adaptation of the institutions at Nell Lane, Withington, and Crumpsall for the reception of patients who can pay three guineas a week is all that has happened up to now, or whether patients have actually been admitted. As it is doubtful that £3 3s. a week can cover the maintenance cost, an entirely new form of addition to the rates is probably involved, and the Ministry of Health is wise to exercise its supervisory powers. At Devonport the Guardians' Infirmary Committee have brought forward proposals similar in character to the Manchester scheme, in that they provide for the reception of members of the general public who cannot wait for voluntary hospital beds, but nothing is said about the cost, except that it is hoped that the Town Council will meet the expense. Co-operation with local voluntary hospitals has been considered and pronounced, for undisclosed reasons, impracticable.

### MEDICAL REPRESENTATION.

As a possible solution of the great difficulty in finding one body competent to speak for the medical profession as a whole, the *Lancet* urges that collective medical opinion should be based upon some adequate expression of the voice of medicine locally. Therefore, it is held, the local medical committees statutory under the Insurance Acts might be readily made representative in their own areas. Also that medical organisations should exert their influence locally through these committees, from which a central executive might be elected. The scheme is, on the face of it, a good one, and offers, we think, far greater possibilities than that foreshadowed in the B.M.A.'s recent memorandum on its attempts to satisfy the requirements of the Ministry of Health. On the other hand, in the broader aspect of this problem—namely, general health administration—it is difficult to imagine a more suitable opportunity than that provided by the Federation of Allied and Medical Societies, in which each body can retain its own individuality and yet adequately express its own views. It seems, indeed, as though a happy combination might be obtained by development of our contemporary's proposals, whose pictured central executive might well co-operate with its sister professions under facilities such as the Federation provides.

### SUDDEN DEATH OF A HOSPITAL WORKER.

The sudden death of Mr. Alfred Fletcher, financial secretary to the Royal Victoria Infirmary, Newcastle-on-Tyne, has come as a great shock to his numerous friends, especially to those who were his colleagues in the infirmary. He came to London about the middle of last month to advise on the possibility of initiating a movement on lines similar to those which had proved so successful under his organisation in Newcastle. He was attacked by pneumonia within three days, and despite every care died at St. Thomas's Hospital on February 23. It was mainly due to Mr. Fletcher that the Royal Victoria Infirmary was able during the five years of war to make income meet expenditure and his death has occurred at the zenith of his success, for the record of the past year, now in the Press, shows it to be the most successful so far as his work is concerned. He was a man of great energy and a master of method. His enthusiasm for his work never flagged and his system enabled him to retain a firm grasp on all the sources of support in the area served in the hospital. At all times and in all weathers he preached the Royal Victoria Infirmary and its work. He was a familiar figure at the colliery, at the dinner-hour in the shipyard, to the night-shift men, at the Miners' Lodge, in the village institute, in the cattle mart, in the parish hall. When he was appointed secretary to the Subscriptions Committee in 1906 the subscribed income of the Infirmary amounted to £14,000, of which £7,000 was derived from workmen's contributions. The subscribed income in 1920 amounted to £89,000, of which £45,000 was raised from the working men. When the work of the hospital was reorganised Mr. Fletcher was appointed financial secretary as well as secretary to the Subscriptions Committee, and he gave material assistance to the Committee formed to raise money for the building of the Orthopædic extension to the Royal Victoria Infirmary, which in eighteen months raised a sum of £150,000. He was a man of wide interests and he gave his best to all he undertook. He was deeply interested in the adult school movement and was an active member of the United Methodist Church. He will be greatly missed in Newcastle where his work brought him into close touch with every form of human activity in the district.

### MORE STANDARDISATION.

If the proposed Vaccines, etc., Standardisation Act really implies the development of a large and entirely new scientific department under Government control, it is a little difficult to see that circumstances call for such provision. If, on the other hand, legislation is intended merely to ensure that those laboratories and manufacturers producing these substances conform to a series of standards such as those set for the majority of drugs, then the situation is more understandable. Nevertheless, the General Medical Council, which body is responsible for the British Pharmacopœia, might well have dealt with this matter also. In any case, it seems that a Government Department with laboratories complete will have considerable amount of investigational work to get through before it is in a position justifiably to direct or criticise the operations of many of the trusted and excellent testing institutions which have now been in existence for years. It seems that the Government would be better advised to obtain their co-operation, than attempt to control this highly specialised work.

### THE DANGEROUS DRUGS ACT.

The Home Secretary continues to answer a multitude of questions with regard to the Dangerous Drugs Act, although, as claimed by him, the preliminary draft received the blessing of the Royal Colleges of Physicians and Surgeons, apart from certain minor suggestions, and the General Medical Council has approved the Regulations, with the reservation that directors of research laboratories might be included among the classes of persons authorised to be in possession of the drugs. The Pharmaceutical Society of Great Britain, replying to the Minister's inquiry, held that the Regulations to prohibit the sale of the drugs to the public except on a medical prescription was *ultra vires* of the Act, and suggested the desirability of separating the functions of prescribing and dispensing. Mr. F. A. Hocking, of the London Hospital, has informed *The Pharmaceutical Journal* that a special scheme for hospitals is being evolved, although he is not at liberty to state details; but Mr. Billington, Hon. Secretary to the Public Pharmacists' Association, sends to the same journal some special regulations for hospitals prepared by his committee. It is not clear if Mr. Hocking's and Mr. Billington's schemes are one and the same, and we defer giving details of the latter. In reply to the eighteen organisations interested in the draft Regulations who ask for the appointment of a representative committee to overhaul them, the Home Secretary considered that the appointment would cause much delay and would not be likely to lead to any useful result.

### "SLEEPY SICKNESS."

The Health Ministry's official memorandum on the occurrence of this disease in Great Britain has served not only to allay a certain amount of press-provoked alarm, but has given practitioners a useful statement of certain now established facts. In brief, these amount to recognition that encephalitis lethargica is due to a filterable virus, and that the disease has been experimentally communicated to animals. Also that the virus is contained in the blood, cerebral tissues, and nasal mucous membrane of infected persons. There have been further steps, however, than the Ministry records. The virus is claimed to have been obtained in artificial culture, and to be a minute organism closely resembling the "globoid bodies" described by Flexner and Noguchi in cases of poliomyelitis. It is less than $\frac{1}{1000}$ of an inch in diameter, and can be distinctly visible under an oil immersion lens. On these points there is considerable, if not complete, scientific agreement, and it is found also that infection can occur through the nasal mucous membrane only when its continuity is previously broken. It is again exactly parallel to epidemic poliomyelitis, and, apart from its claims as a piece of interesting special pathology, this work must also be viewed as a decided step in technique for dealing with the so-called ultra-microscopic organisms, to which type the causal germs of a number of common, but as yet partially understood, infectious diseases apparently belong.

### A GREAT ANNUAL MEETING.

The annual meeting of the centenary year of the Seamen's Hospital Society held on March 1 bids fair to outdo the records of the famous annual meetings of this Society of recent years. As Admiral Sir Henry Jackson said in his speech, the work of merchant seamen has gone on for centuries, but it is only their heroic conduct in the war that has brought home to the public the wonderful courage and bravery of these men of the Red Ensign, and whatever is done for them is only scant justice in return for what they have done for the Empire. H.R.H. the Prince of Wales presided at the meeting and must have been gratified at the size of the gathering. The Grand Hall of the Hotel Cecil was filled even to its galleries long before the hour of the meeting and at its conclusion an overflow meeting was held. A distinguished company assembled for the principal meeting, at which Sir Perceval Nairne, Lord Inchcape, Capt. Sir Arthur Clarke, Admiral Sir Henry Jackson, Capt. Sir H. Acton Blake, and Mr. J. Havelock Wilson spoke. The Prince of Wales appealed for equal help for our sick merchant seamen in peace as in war, and especially for the necessary funds for the sanatorium now approaching completion for men suffering from tuberculous disease. £100,000 was asked for and £90,000 is in hand. As a result of the Society's wise and conservative financial policy its group of hospitals stands on a thoroughly sound basis. All will echo the Prince's hope that in this the one hundredth year of the Society's existence the greatest generosity may be shown in the cause of our sick seamen. The Dreadnought is known and loved by sailors and seamen all over the world, and the British public even in these difficult times is not likely to hold back from the support of a charity with such a reputation.

### THE BRIGHTON PROVIDENT SCHEME.

Dr. Gordon Dill explained the provident scheme for hospital benefits, in the Brighton neighbourhood, to a recent meeting of the Oratory Club, and stated that the scheme is going very well. Dr. Dill claimed that the Association that has been formed takes its members out of the sphere of charity altogether, because they pay collectively the whole cost of the maintenance of any member who may require hospital treatment —assuming only that the incidence of sickness in any one year is not above the ordinary. Those interested in provident plans will observe the progress of the Brighton scheme with interest. We are rather inclined to agree with one of the speakers at the meeting who said that the only danger is that the provident organisation may become too successful and make larger demands on the hospitals than they can cope with. But this may mean only temporary inconvenience leading to enlargement schemes which would not have been made possible by other means.

### THE DENTISTS BILL.

It is not surprising that the Dentists Bill has been criticised. With so many features that the qualified dentist on the present Register must inevitably resent, there will probably be still further protest. The first objective of the Ministry should be improved public welfare and health, and a better dental service for the masses is urgently required, but, as we have previously urged, if public sympathy is to be obtained for the apparent admission of a host of "unqualified" people to the ranks of this service, then the originators must more clearly and publicly state the precise facts of the situation, the safeguards, and the exact reasons why their measure is held to foreshadow a great public benefit.

### FOOTBALL HOSPITAL SATURDAY.

Early in the football season the great clubs have what are called "practice" matches, and as the Football Association will not allow the gate-money to be ear-marked for club purposes it often happens that hospitals and other charitable institutions benefit. These practice matches are very attractive to the public, and as many as 20,000 spectators will congregate on a favourable afternoon on a popular ground such as that at Chelsea. It has been suggested that it might be possible to enlarge upon the idea and to organise a general movement throughout the country in the shape of a Football Hospital Saturday. It is thought that the players throughout the country would give their services free, and if local teams could be matched against each other, such as Newcastle and South Shields, Hull City and Grimsby, the travelling expenses would not be heavy. The *Daily Mail* asks, Who is the football magnate who will earn fame for himself by instituting a Football Hospital Saturday?

### A COURAGEOUS COTTAGE HOSPITAL.

Nothing is more cheering than the prosperity of our cottage hospitals, but few in these days can record such a balance as that of £831, which stands to the credit of the Clacton and District Cottage Hospital. The balance, of course, is merely a resource already devoted, for improvement and extension are the only profits which a voluntary hospital exists to show. The Clacton district is growing in population, patients are increasing, and therefore the hospital has done well to purchase a piece of land for £600. Mr. E. Weldon, the hon. secretary, thinks the land not only a sound investment but a good bargain, whereby the hospital's capital, as Dr. A. E. Cowley well put it, is being used to the best advantage of the institution.

### UNEMPLOYMENT AND MAINTENANCE CHARGES.

After six months' experience of an appeal to patients for voluntary contributions according to their means, the Royal Bucks Hospital is seriously considering "compulsory payments," which means, we take it, charges toward the cost of maintenance. The plan seems to have alarmed the Friendly Societies, who have been invited to confer again with the Committee before a renewed discussion to be held on April 6 next. The charges proposed are, for in-patients 10s. 6d. to 35s. per week, or their daily equivalent fraction; but treatment free of the above charges will be given to all those who make regular weekly contributions. For adults 1d. is suggested; for a married man 2d., and for a family man with any children under sixteen a sum of 3d. would ensure full treatment for all. An interesting clause is: "Should a subscriber become unemployed and require treatment, he shall be entitled to same during the term of unemployment, without payment for the remainder of the current year." Let the Friendly Societies be equally frank and state their case publicly, if only to avoid any suspicion that it would not bear open expression.

### THIS WEEK'S DRUG MARKET.

The downward movement of prices has apparently not reached its end; indeed it seems to have become more comprehensive. An unexpected and substantial reduction has been made in quotations for bismuth carbonate, bismuth nitrate, and other salts of bismuth, following on a fall in the price of the metal. English makers have reduced their quotations of potassium iodide in order to meet foreign competition; prices of iodide, iodoform, and sodium iodide, however, are unchanged. Consequent upon the continued good results of the Norwegian cod fishing the price of cod-liver oil has again receded and the general opinion is that this will fall still further. Among other articles which are cheaper may be mentioned honey, senna, camphor, cloves, clove oil, lemon oil, bergamot oil, star aniseed oil, phenazone, thymol, and cream of tartar, while a number of synthetic products still have a downward tendency.

